# Physician Electronic Health Record Use After Changes in US Centers for Medicare & Medicaid Services Documentation Requirements

**DOI:** 10.1001/jamahealthforum.2023.0984

**Published:** 2023-05-12

**Authors:** Natalya Maisel, Robert Thombley, J. Marc Overhage, Kathleen Blake, Christine A. Sinsky, Julia Adler-Milstein

**Affiliations:** 1 University of California, San Francisco, San Francisco; 2 Overhage Group, Indianapolis, Indiana; 3 American Medical Association, Chicago, Illinois

## Abstract

This cohort study examines changes in physician electronic health record (EHR) documentation time before and after changes in Centers for Medicare & Medicaid evaluation and management requirements.

## Introduction

On January 1, 2021, the US Centers for Medicare & Medicaid Services (CMS) modified outpatient evaluation and management (E/M) coding requirements, including the elimination of history and physical examination documentation. Centers for Medicare & Medicaid Services sought to reduce physician documentation burden^1^ by reducing electronic health record (EHR) documentation time. This study assesses changes in outpatient physician documentation time after these changes.

## Methods

The national sample for this cohort study included all health care organizations using the Cerner Lights On Network (Oracle) EHR (n = 196) from September 2020 through December 2021. The University of California, San Francisco Institutional Review Board approved the study and waived the requirement for informed consent because the data were deidentified. This study followed the STROBE reporting guidelines.

We limited analyses to 4 specialties with varied E/M volume: family medicine (high volume), internal medicine (high), cardiology (moderate), and orthopedics (low). We assessed active EHR notes documentation time based on frequent software, keyboard, and mouse actions in minutes per visit^2^ (eMethods in Supplement 1).

First, we calculated weekly averages by specialty and longitudinal trends in documentation time per visit. Next, we replicated and extended analyses by Apathy et al^3^ (completed using a Signal [Epic Systems Corp] EHR) by comparing documentation time across 3 periods: period 1, end of 2020 (September-December 2020; before the E/M changes); period 2, immediately after the E/M changes (January-April 2021); and period 3, end of 2021 (September-December 2021, 1 year after period 1) to control for seasonal effects and to test longer-term associations (eg, learning curve). In addition to paired *t* tests, we constructed multilevel mixed-effects models to control for visit volume (eMethods in Supplement 1). We also assessed physician-level variability in response to the E/M changes. A 2-sided *P* value <.05 was considered statistically significant. Data were analyzed using Stata, version 17.0 (Stata Corp).

## Results

The sample included 18 392 physicians and 876 832 physician-weeks. Documentation time was decreasing slightly before the E/M changes, and this trend continued after the changes (Figure). In periods 1 and 2, internal medicine, cardiology, and orthopedic physicians had no change in mean documentation time, while family medicine physician documentation time increased by 4.8 seconds (Table). When we compared periods 1 and 3, mean documentation time was significantly lower for all 4 specialties: internal medicine (16.2 seconds), family medicine (13.8 seconds), cardiology (12.6 seconds), and orthopedics (6.6 seconds) (Table). Controlling for visit volume, for all specialties, there was a significant increase in documentation time per visit from periods 1 to 2, and a significant (but smaller magnitude) decrease from periods 1 to 3.

**Figure.**
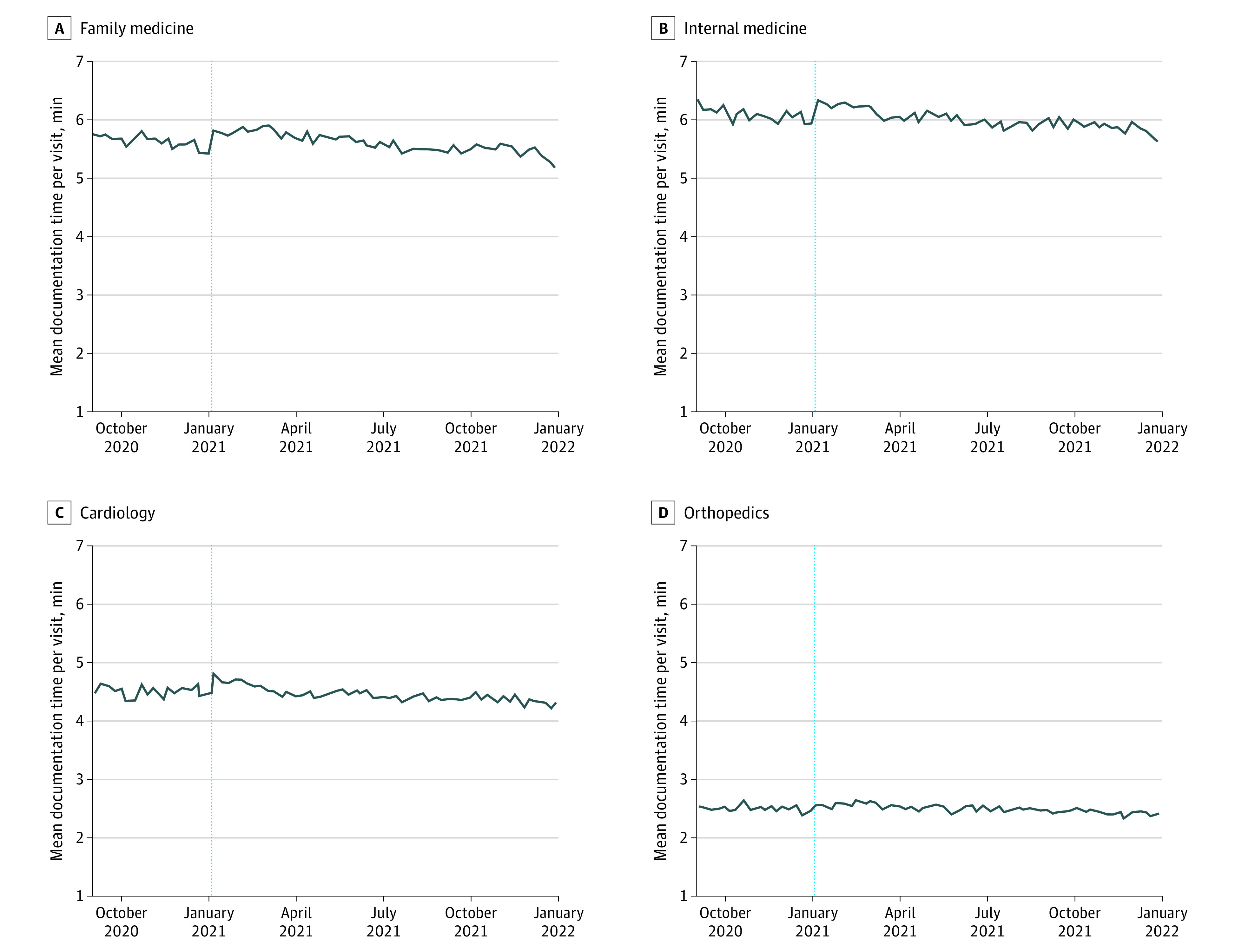
Physician Time Spent in the Electronic Health Record (EHR) on Documentation Per Visit Vertical lines represent January 1, 2021, when evaluation and management coding changes were implemented. Data represent physician-weeks with at least 5 visits and any EHR time. Outlier physician-weeks with visits above the 99th percentile for each specialty (mean 148.3 visits) or EHR minutes per visit above the 99th percentile for each specialty (mean 57.32 minutes) are excluded. Mean (SD) physician week-level overall EHR time by specialty was 18.17 (10.31) minutes for family medicine, 19.53 (12.46) minutes for internal medicine, 11.80 (8.32) minutes for cardiology, and 6.02 (4.52) minutes for orthopedics.

**Table.** Physician Time Spent in the Electronic Health Record (EHR) on Documentation Per Visit Before and After Evaluation and Management Coding Requirement Changes

Specialty	Mean (SD) No. of weekly visits per period^a^			Documentation minutes per visit, mean (SD) (n = 18 392 physicians)^b^			Estimated mean difference (95% CI)			
	Period 1	Period 2	Period 3	Period 1	Period 2	Period 3	Periods 1 vs 2^c^		Periods 1 vs 3^c^	
							Minutes	Seconds	Minutes	Seconds
Family medicine	44.27 (24.66)	48.27 (26.54)	48.51 (26.65)	5.66 (4.05)	5.75 (4.11)	5.43 (3.87)	0.08 (0.05 to 0.12)	4.8 (3.0 to 7.2)	−0.23 (−0.28 to −0.19)	−13.8 (−16.8 to −11.4)
Internal medicine	26.90 (22.63)	29.50 (25.12)	28.62 (24.26)	5.99 (4.90)	5.99 (4.87)	5.72 (4.67)	0.01 (−0.05 to 0.06)	0.6 (−3.0 to 3.6)	−0.27 (−0.33 to −0.20)	−16.2 (−19.8 to −12.0)
Cardiology	23.17 (20.26)	25.99 (23.39)	25.69 (22.64)	4.39 (3.96)	4.40 (3.99)	4.18 (3.91)	0.00 (−0.04 to 0.05)	0.0 (−2.4 to 3.0)	−0.21 (−0.27 to −0.15)	−12.6 (−16.2 to −9.0)
Orthopedics	22.50 (21.97)	24.73 (24.59)	25.43 (24.88)	2.53 (2.44)	2.53 (2.39)	2.43 (2.32)	0.00 (−0.04 to 0.03)	0.0 (−2.4 to 1.8)	−0.11 (−0.15 to −0.06)	−6.6 (−9.0 to −3.6)

^a^ Period 1, before change (September-December 2020); period 2, immediately after change (January-April 2021); period 3, 1 year after change (September-December 2021).

^b^ To replicate Apathy et al,^3^ data are presented as physician-level means per period aggregated from physician-week data. All physicians included in the study had at least 1 week of data in each of the 3 periods.

^c^ To replicate Apathy et al,^3^ data are calculated using physician-level paired *t* tests.

Finally, as for physician-level variability in documentation time from periods 1 to 3, 56.7% of family medicine physicians, 54.9% of internal medicine physicians, 56.9% of cardiology physicians, and 53.9% of orthopedic physicians decreased documentation time per visit.

## Discussion

Across the 2 largest EHR vendors in the US, this study, along with that of Apathy et al,^3^ found small reductions in documentation time following the changes in CMS E/M coding requirements, but not at clinically meaningful levels. Apathy et al^3^ observed a small decrease immediately postimplementation, while reductions took longer to manifest in the Cerner EHR sample. The magnitude of reduction was modest in both studies and less than the 19-second CMS-estimated reduction in documentation time per visit.^4^

Study limitations include a focus on 1 EHR vendor and our inability to distinguish time spent on different documentation domains. It is possible that reductions in documentation in the domains affected by the CMS regulations freed up time that physicians used for higher-value documentation in other domains, which could explain the sizeable physician-level variation we found. Even if total documentation time is not dramatically reduced, the scaled-back E/M requirements could reduce physicians’ cognitive burden and improve their work experience.^5^

## Notes

Supplement 1.
eMethods.

Supplement 2.
Data Sharing Statement
