# Errors in the Discussion Section and References

**DOI:** 10.1001/jamahealthforum.2023.1917

**Published:** 2023-06-16

In the Research Article titled “Physician Electronic Health Record Use After Changes in US Centers for Medicare & Medicaid Services Documentation Requirements”^1^ published online on May 12, 2023, the last sentence in the first paragraph of the Discussion section and reference 4 were incorrect. The sentence in the Discussion section should be “While the magnitude of reduction is modest in both studies, it is consistent with the projected impact of the regulations (CMS referenced an estimated 7-second reduction in documentation time per visit).” The CY year, regulation number, and URL were updated in reference 4. This article has been corrected.
