# Supplementary material for: Physician Electronic Health Record Use After Changes in US Centers for Medicare & Medicaid Services Documentation Requirements
Source: JAMA Health Forum. 2023 May 12;4(5):e230984. doi: 10.1001/jamahealthforum.2023.0984 (PMC10182425; doi:10.1001/jamahealthforum.2023.0984)
Supplement: Supplement 1. — eMethods. [file jamahealthforum-e230984-s001.pdf]

Supplemental Online Content

Maisel N, Thombley R, Overhage JM, Blake K, Sinsky CA, Adler-Milstein J. Physician electronic health record use after changes in US Centers for Medicare & Medicaid Services documentation requirements. *JAMA Health Forum.* 2023;4(5):e230984. doi:10.1001/jamahealthforum.2023.0984

eMethods.

This supplemental material has been provided by the authors to give readers additional information about their work.

© 2023 Maisel N et al. *JAMA Health Forum*.

eMethods

This study followed the STROBE reporting guideline and received human subjects approval from the UCSF IRB. Our analyses focused on 70 weeks of data from Sept 1, 2020 to Dec 31, 2021. There were 2 weeks where the vendor had technical issues that resulted in a loss of records, and those weeks were dropped from our analyses (weeks 36 and 48). Our final dataset included physicians in one of the 4 specialties of interest (Family Medicine, Internal Medicine, Cardiology, Orthopedics) with data for at least 1 week in each of the 3 time periods tested in our main analyses (Sept-Dec 2020, Jan-April 2021, Sept-Dec 2021).

We conducted paired t-tests to obtain estimated mean differences and 95% confidence intervals. For our multilevel (weeks nested within physician nested within health system) mixed-effect models, we utilized the “mixed” command in Stata version 17.0 to obtain coefficients, standard errors and 95% confidence intervals, with random-effects parameters for physician and client-level effects.

Our main study outcome was the time spent on electronic health record (EHR) documentation per ambulatory visit. EHR time was calculated based on the methodology outlined for Cerner Lights on Network calculations in Overhage and McCallie (2020). Overhage and McCallie (2020) conducted an analysis of the granular specific software modules and services executed by the physician, as recorded in the log entry patterns of the EHR metadata. They then mapped these patterns to clinical tasks, such as responding to alerts, placing orders, and writing documentation, and were able to define a set of activity records based on these tasks (see Table 1 in Overhage and McCallie [2020] for the full list).

© 2023 Maisel N et al. *JAMA Health Forum*.

EHR “active time” was measured with a 2-tiered system. First, if a user was logged in and activity records were fewer than 45 seconds apart, it was considered “active” use. Second, if there was more than 45 seconds between activity records, then mouse clicks, mouse movement, and keystrokes were monitored (see Overhage and McCallie [2020] for exact values used). These movement measurements were based on direct observation of a sample of 337 physicians.

Documentation time spent in the EHR was a subset of overall EHR time and was defined as the active time in the EHR that the physician spent on clinical notes (e.g., creating, editing). In order to standardize documentation time by patient volume, we divided total documentation time during the 7-day week by the number of ambulatory patient visit charts closed in same period. This became our outcome of “documentation time per visit” used as the main dependent variable.

© 2023 Maisel N et al. *JAMA Health Forum*.
