# Supplementary material for: Physician Electronic Health Record Use After Changes in US Centers for Medicare & Medicaid Services Documentation Requirements
Source: JAMA Health Forum. 2023 May 12;4(5):e230984. doi: 10.1001/jamahealthforum.2023.0984 (PMC10182425; doi:10.1001/jamahealthforum.2023.0984)
Supplement: Supplement 2. — Data Sharing Statement [file jamahealthforum-e230984-s002.pdf]

Data Sharing Statement

Maisel. Physician Electronic Health Record Use After Changes in US Centers for Medicare & Medicaid Services Documentation Requirements. *JAMA Health Forum*. Published May 12, 2023. doi:10.1001/jamahealthforum.2023.0984

Data
Data available: No

Additional Information
Explanation for why data not available: Data used in this study are subject to a DUA with Cerner that does not allow them to be shared outside UCSF.
